# HD‐DRUM Rhythmic Training in Huntington's Disease: Feasibility, Motor, and Brain Microstructural Pilot Signals

**DOI:** 10.1002/mdc3.70871

**Published:** 2026-10-23

**Authors:** Pedro Luque Laguna, Vasileios Ioakeimidis, Cheney J.G. Drew, Philip Pallmann, Monica Busse‐Morris, Guy B. Watson, Robin Schubert, Anne E. Rosser, Claudia Metzler‐Baddeley

**Affiliations:** ^1^ Cardiff University Brain Research Imaging Centre (CUBRIC), School of Psychology, Cardiff University Cardiff UK; ^2^ Danish Research Centre for Magnetic Resonance, Department for Radiology and Nuclear Medicine Copenhagen University Hospital Amager and Hvidovre Copenhagen Denmark; ^3^ Centre for Trials Research, School of Medicine, Cardiff University, Neuadd Meirionnydd Cardiff UK; ^4^ Faculty of Nursing, Midwifery and Palliative Care, King's College London UK; ^5^ Huntington's Disease Association—HD Voice Public and Patient Input Liverpool UK; ^6^ George‐Huntington‐Institute Muenster Germany; ^7^ Department of Psychological Medicine and Clinical Neurosciences School of Medicine, Cardiff University Cardiff UK; ^8^ Cardiff Brain Repair Group Lifescience Building (BIOSI 3) Cardiff UK

**Keywords:** brain microstructure, Huntington's disease, paced tapping, randomized controlled feasibility trial, rhythmic training

## Abstract

**Background:**

Rhythmic cuing may benefit motor functions in Huntington's disease (HD) by compensating for the loss of predictive timing associated with basal ganglia degeneration. To our knowledge no remote rhythmic training application has been developed or evaluated that seeks to improve function in HD. HD‐DRUM is a tablet‐based rhythmic cuing application for the training of paced movements. Feasibility of 8 weeks at‐home HD‐DRUM compared with usual‐activity control was assessed in a two‐arm, pilot randomized controlled trial (RCT) in people with HD. HD‐DRUM‐related changes in motor and cognitive functions, mood, and brain microstructure were explored.

**Objectives:**

Primary outcomes were feasibility rates (recruitment, retention, adherence, acceptability) for an effectiveness RCT. Secondary outcomes were estimates of HD‐DRUM‐related effect sizes on motor functions, cognition, mood, and brain microstructure to explore training mechanisms.

**Methods:**

Fifty‐five individuals with HD were randomized into either HD‐DRUM (n = 32, 1 excluded) or control (n = 23) groups. Participants underwent Quantitative‐Motor, cognitive and mood assessments and diffusion‐weighted MRI at baseline and follow‐up. Recruitment, retention, and adherence rates were recorded, and HD‐DRUM acceptability was assessed with a self‐report questionnaire. Group differences of changes in behavioral and brain microstructural measures were reported with 95% confidence intervals.

**Results:**

Acceptability of HD‐DRUM, retention, and adherence rates averages were high (>80%) and target recruitment was reached. Performance in paced tapping and brain microstructure in motor networks signaled training benefits and were associated with each other.

**Conclusions:**

HD‐DRUM may provide an accessible intervention that can aid maintenance of motor function and brain microstructure in HD.

Huntington's disease (HD) is an inherited neurodegenerative disease, characterized by the progressive decline in cognition, movement, and mood. Striatal atrophy^1^ and white matter alterations^2^ can be detected many years before disease onset, leading to clinical symptoms^3^, ^4^, ^5^ with debilitating effects on a person's quality of life.^6^

There are no licensed disease‐modifying treatments for HD. Although novel therapeutic approaches are under investigation, including gene and cell therapies, with the potential to slow disease progression,^7^, ^8^ many are complex to deliver, costly, and unlikely to be accessible to all patients. Symptomatic pharmacological treatments are typically limited in effectiveness and often associated with significant side‐effects.

HD mouse model studies have shown that environmental enrichment can slow disease progression, rescue protein deficits,^9^, ^10^, ^11^ and improve morphology and function of striatal grafts.^12^, ^13^ Thus, behavioral or lifestyle interventions have the potential to help with the management of HD disease progression.

Music‐based Rhythmic Auditory Stimulation (RAS) uses external cues to facilitate movement^14^ and may improve gait and mobility in people with Parkinson's disease (PD).^15^, ^16^, ^17^ Although PD and HD are clinically distinct, both involve basal ganglia (BG) neurodegeneration and impairments in response timing and rhythm generation.^18^ The BG, together with auditory and motor cortical regions, form networks that support beat perception and rhythm processing^19^, ^20^: the putamen is thought important for generating temporal predictions, the Rolandic operculum for sensorimotor integration, and precentral sulcus and supplementary motor area (SMA) for motor planning and control.^21^ RAS may compensate for impaired BG timing signals in PD and HD through external rhythmic cueing.^14^ However, the neural mechanisms and clinical effects of RAS in HD remain largely unexplored.

Based on pilot work on Bongo drumming in people with HD,^22^, ^23^ we designed a tablet‐based drumming application, HD‐DRUM,^24^ which involves the learning of rhythmic patterns through tapping, while measuring performance and adherence. As a first step in evaluating HD‐DRUM as a therapeutic intervention, we conducted a two‐arm pilot RCT. The primary objective was to assess the feasibility of a larger effectiveness trial by examining recruitment, retention, adherence and acceptability of 8‐weeks HD‐DRUM training. Secondary objectives were to estimate effect sizes (ES) associated with changes in cognitive and motor performance and MRI‐derived microstructural measures in brain regions critical for rhythmic processing.

## Methods

The study received ethical approval from the Wales Research Ethics Committee 2 (REC reference: 22/WA/0147), was sponsored by Cardiff University (SPON1895‐22), and prospectively registered with ISRCTN 11906973 (https://doi.org/10.1186/ISRCTN11906973). The design was a multi‐site two‐arm unblinded pilot RCT with parallel HD‐DRUM intervention and usual‐activity control groups. The full protocol has previously been published.^25^

### Participants and Study Setting

Participants were identified, approached, and recruited at five HD clinics, that participate in the observational study Enroll‐HD^26^ in the UK (Cardiff, Liverpool, Exeter, Bristol, Birmingham). They gave written informed consent prior to participating in the study.

### Inclusion Criteria

Individuals over the age of 18 years with HD confirmed by genetic testing for the presence of a CAG repeat length ≥36 in the huntingtin gene and a Unified Huntington's Disease Rating Scale (UHDRS)^27^ Total Functional Capacity (TFC) score between 9 and 13 (normal function to mild functional impairment).

### Exclusion Criteria

A history of any other major disabling neurological or neurodegenerative condition such as Alzheimer's disease, Parkinson's disease, brain tumor, or stroke. MRI contra‐indications precluded MRI assessment but not participation in the trial.

### Study Procedure

Participants completed baseline cognitive, motor, mood, and MRI assessments at the Cardiff University Brain Research Imaging Centre (CUBRIC) before being randomized to the HD‐DRUM or control group, stratified by UHDRS‐TFC score to balance disease burden.^25^ HD‐DRUM participants received a Samsung Galaxy 8 tablet with the app and instructions for home use, with researcher support to promote adherence. Control participants continued their usual activities without additional contact. After 8 weeks, all participants were invited to a follow‐up appointment at which the baseline assessments were repeated. HD‐DRUM participants also completed an acceptability questionnaire. Control participants were offered access to HD‐DRUM after completing the study.

### HD‐DRUM Intervention

Details of the HD‐DRUM content and delivery have been published.^24^ HD‐DRUM comprises 22 audio‐guided training sessions introducing rhythmic patterns with and without metronome or musical background. Participants tapped along to rhythms with the left hand on a blue triangle and the right hand on a red circle on the tablet, receiving visual and auditory feedback. Tempo and complexity increased across sessions, with practice progressing from unimanual to bimanual tapping. Participants could repeat sessions as desired but needed to achieve 60–70% response accuracy to progress to the next level. Response speed, accuracy, and training frequency and duration were recorded. The instruction manual recommended 10–15 minutes of practice, 5 times per week for 8 weeks (40 sessions in total).

### Primary Feasibility Outcome Measures

*Recruitment* was assessed as by the extent to which the target sample size of 50 was achieved. *Retention* was defined as the proportion of recruited participants who completed the study with reasons for withdrawal and loss to follow‐up recorded where possible.

*Adherence* was monitored within HD‐DRUM by recording training frequency and duration. Adherence by frequency was calculated as the percentage of days completed (a day with ≥5 minutes of practice was counted as meaningful engagement) relative to the prescribed 40 days (5 days/week for 8 weeks). Adherence by duration was calculated as the percentage of total training minutes relative to the expected dose (451 minutes; 40 sessions × 11.3 minutes average session duration). These measures allowed the separate evaluation of adherence to recommended frequency and duration of engagement.

*Acceptability* was assessed using a self‐report questionnaire covering enjoyment, engagement, ease of use, perceived effects, and suggestions for improvements (Supplementary File, Supplemental Table 1). Overall acceptability was defined as the proportion of participants who agreed with the first statement “I enjoyed using the app.”

Feasibility success criteria were predefined using a traffic light system: green (all metrics ≥70%) signaling trial success, amber (no metric <40% but at least one <70%) the need for further process evaluation; and red (at least one metric <40%) that a future RCT would not be feasible.

### Secondary Outcome Measures

Cognitive, motor, and mood assessments were conducted at baseline and follow‐up in a quiet testing room and lasted approximately 2 hours, including breaks.

#### Cognitive Function

Executive function^28^ was assessed with paper‐and‐pencil and computerized tests (from the Psychology Experiment Building Language (PEBL) battery^29^ and MATLAB^30^) including Stroop,^31^ verbal trails,^32^ verbal fluency,^33^ Symbol Digit Modality Test (SDMT),^34^ PEBL‐Eriksen flanker,^35^ and MATLAB‐based n‐back paradigms.^36^, ^37^ Thirty‐one cognitive variables were selected for analyses, comprising total correct responses (Stroop, verbal fluency, SDMT), response times (flanker, Stroop, verbal trails), and the sensitivity index A'^38^ for n‐back performance.

#### Motor Function

Motor performance was assessed with the Quantitative Motor (Q‐Motor) test battery,^39^ including speeded and metronome‐paced tapping of left and right index fingers and feet, and dual finger pointing‐and‐tapping tasks. Metronome tapping was performed at slow and fast tempi, with and without auditory cue. For each condition, mean and standard deviation (SD) were computed for tapping speed (inter‐onset interval, IOI; s), force production (area under the force–time curve, AUC) and pacing accuracy (absolute deviation from target tempo). Additional kinematic measures were extracted for speeded and pointing tasks.^25^, ^39^ This yielded 96 measures for metronome tapping, 72 for speeded tapping and 22 measures for the pointing‐and‐tapping task per session.

#### Mood

Mood was assessed with the Patient Health Questionnaire (PHQ‐8).^40^

#### MRI Brain Microstructure

Multi‐shell high angular resolution diffusion imaging^41^ data were acquired on a 3 T Siemens Connectom system with strong (300mT/m) gradients^42^ using single‐shot spin‐echo EPI (TR/TE = 3000/59 ms; 2 mm isotropic; GRAPPA = 2; AP phase‐encoding). Diffusion weighting comprised b‐values of 200, 500, 1200, 2400, 4000 and 6000 s/mm^2^ (20, 20, 30, 61, 61 and 61 directions, respectively), with 15 b = 0 s/mm^2^ images (13 AP interleaved plus 2 PA) (~18 min acquisition time). Additional myelin‐sensitive sequences (quantitative magnetisation transfer,^43^ multicomponent relaxation with steady‐state imaging^44^) were acquired but are not reported here.

Diffusion data were pre‐processed using MRtrix3^45^ and FSL 6.03, including Marchenko–Pastur PCA denoising,^46^ Gibbs‐ringing correction,^47^ brain masking, susceptibility‐induced distortion correction with TOPUP (AP/PA *b* = 0 images),^48^ and motion and eddy‐current distortions correction with slice‐wise outlier replacement using EDDY.^49^, ^50^ Gradient non‐linearity^51^ and MRI signal‐drift corrections were applied using in‐house MATLAB scripts.

Four diffusion models were fitted to derive parametric maps that underwent visual quality control. Diffusion tensor imaging^52^, ^53^ was fitted to the *b* = 1200 s/mm^2^ shell to estimate fractional anisotropy (FA), mean diffusivity (MD), and radial diffusivity (RD). Neurite orientation dispersion and density imaging^54^ was fitted using *b* = 200–2400 s/mm^2^ to derive neurite density index (NDI), orientation dispersion index (ODI) and isotropic signal fraction (ISOSF). The Soma and Neurite Density Imaging model^55^ was applied to all shells yielding neurite and soma signal fractions (fneurite, fsoma), apparent soma radius (Rsoma), and extracellular diffusivity (De). CHARMED^56^ was fit to estimate restricted signal fraction (RF).

Final outcomes were computed by averaging each metric within anatomically defined region of interests (ROIs). Gray matter ROIs were derived using FreeSurfer's longitudinal stream,^57^ yielding bilateral masks for the caudate, putamen, globus pallidus, thalamus, primary motor cortex, and SMA areas (paracentral and superior frontal labels from the Desikan‐Killiany parcellation). Native‐space diffusion maps were projected to each participant's longitudinal T1 template. White‐matter tracts were reconstructed using TractSeg^58^, ^59^ applied to fiber orientation distribution peaks generated in MRtrix3.^45^ Tracts of interest included seven corpus callosum segments,^60^ bilateral corticospinal tracts, and striato‐prefrontal and striato‐premotor tracts. Tract‐average metrics were computed with streamline density weighting. In total, 168 gray‐matter and 156 white‐matter microstructural outcome measures were obtained per participant and session.

### Disease Stage Classification

Participants’ HD‐ISS staging^61^ was determined using the online HD‐ISS calculator.^62^ CAG repeat length, UHDRS^27^ TFC, Total Motor Score, Independent Scale, and SDMT^27^ from participants’ most recent Enroll‐HD assessments were obtained.^26^ These preceded baseline by a median of 57 days [interquartile range (IQR) 34–83] in 48 participants. Three participants had their first assessment after study completion, and three did not participate in Enroll‐HD. Normalized striatal volumes were derived from the study‐acquired T1‐weighted MRI scans.

### Statistical Methods

A pragmatic sample size of 50 participants was targeted, allowing feasibility rates to be estimated with a 95% binomial confidence interval (CI) within ±15 percentage points, irrespective of the observed proportion.^63^

#### Feasibility Rates

Recruitment and retention data were collected and managed using Research Electronic Data Capture (REDCap).^64^ Adherence was recorded in HD‐DRUM. Acceptability was measured via the self‐report questionnaire. Feasibility outcomes were evaluated against above predefined success criteria.

#### Principal Component Analyses

Principal component analysis (PCA) was used to reduce the dimensionality of cognitive, motor, and microstructural outcomes. Percentage change (%Δ) from baseline to follow‐up was calculated for each outcome and separate PCAs were applied to each outcome domain (Supplemental Fig. 1–7). A single PCA was conducted on %Δ of the 31 cognitive variables (Cog‐PC). Four PCAs were conducted on motor outcomes: metronome tapping performance (48 mean variables) (MTM‐PC), metronome tapping variability (48 SD variables) (MTV‐PC), speeded tapping and pointing performance (47 mean variables) (STM‐PC), and variability (47 SD variables) (STV‐PC). Two further PCAs were applied to %Δ*s* of gray‐matter (168 variables) and white‐matter (156 variables) microstructural outcomes (GMM‐PC, WMM‐PC).

To limit over‐extraction in this relatively small sample, component retention followed recommended guidelines,^65^, ^66^ applying the Kaiser criterion (eigenvalue >1) followed by inspection of Cattell scree plots.^67^ Retained components were orthogonally rotated using Varimax with Kaiser normalization,^68^ and component scores of percentage‐changes were calculated for each participant.

#### Estimation of Effect Sizes of HD‐DRUM on Secondary Outcomes

HD‐Drum effect sizes (ES) were estimated using participants’ PCA scores and general linear models (GLMs) for each component, with group as the explanatory variable and age and sex as covariates. Extreme outliers were excluded from each GLM using the Tukey criterion.^69^ Standardized group ES and 95% CIs were displayed using forest plots.^70^ For components where the 95% CI excluded zero, violin plots^71^ aided interpretation of effects, guided by the rotated PCA loading patterns, with variables showing absolute loadings ≥0.50 considered salient.

#### Correlations between Functional and Brain Microstructure Changes

To explore whether any HD‐DRUM‐related changes in cognitive or motor functions were associated with changes in brain microstructure, we conducted correlation analyses between participants’ functional and microstructural change component scores for which the 95% CI of the group effect excluded zero, indicating a positive signal of change. Pearson's and Spearman's correlation coefficients were calculated for each functional‐microstructural component pair, separately for each group. Results were considered exploratory and reported with uncorrected p‐values.

PCAs were conducted in IBM SPSS Statistics (v27).^72^ Descriptive statistics, GLMs, and figures were generated in Python^73^ using *statsmodels* ^74^ and *matplotlib*.^75^

#### Mood

Means and SDs of PHQ‐8 baseline and follow‐up scores were calculated and reported for each group.

## Results

### Recruitment

Fifty‐five participants were recruited, exceeding the target sample size of 50 (Fig. 1, Table 1). One participant was excluded due to a TFC score <9, leaving 54 participants for analyses. Their demographic and clinical characteristics are summarized in Table 2. Groups were balanced for sex and TFC, but control participants were younger in age. HD‐ISS staging was completed in 39 participants; staging was not possible in the remainder due to missing clinical and/or imaging data, CAG repeat length < 40, or undefined staging, i.e., functional before motor impairment.

**Figure 1:**
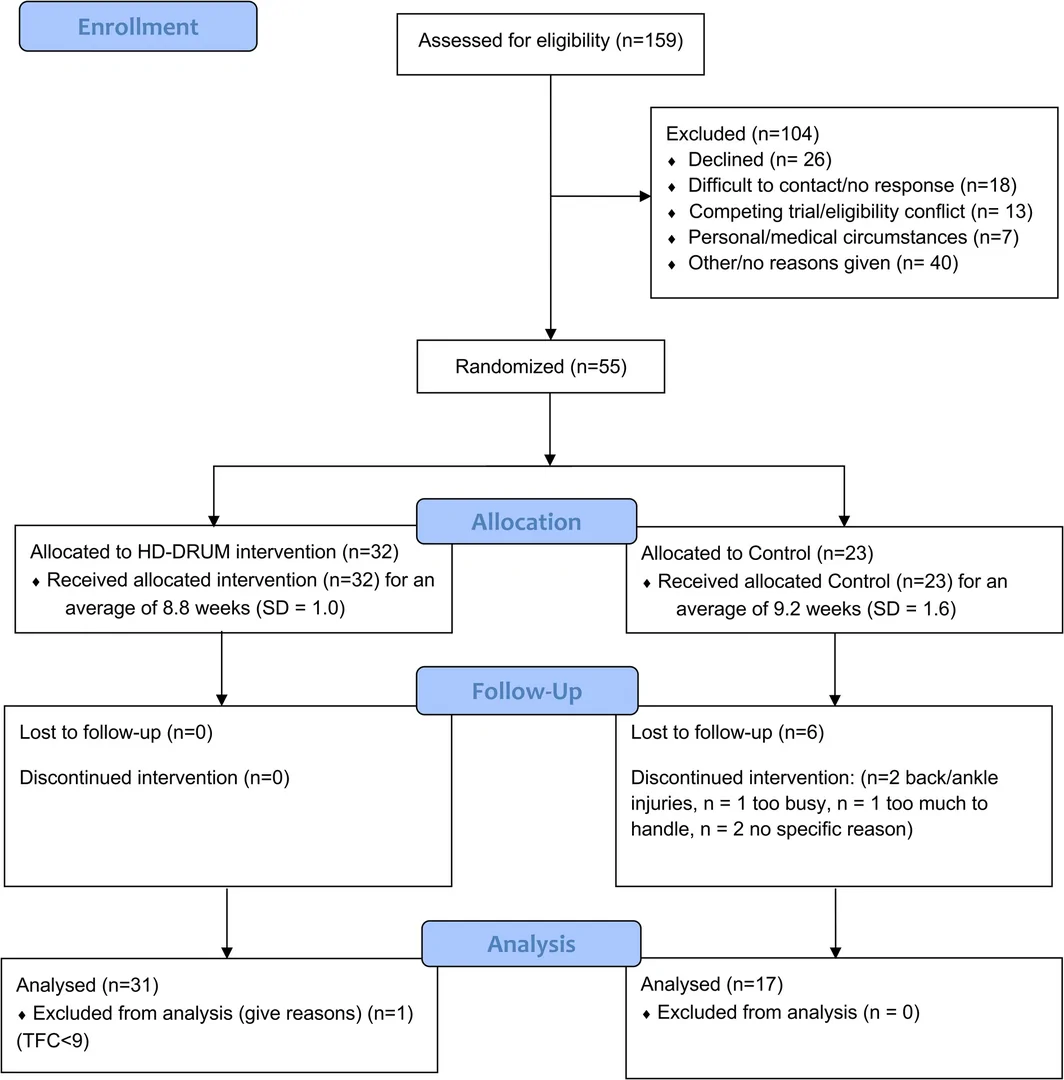
Consolidated standards of reporting trials (Consort) flow chart providing a schematic overview of enrolment, allocation, follow‐up, and analysis stages of two‐arm randomized controlled feasibility trial of HD‐DRUM.

**TABLE 1:** Summary of the feasibility outcomes

	Total HD	HD‐DRUM	Control
Recruited out of approached, n/N (%)	55/156 (34.6%)	32	23
Recruitment of eligible target sample size, n/N (%)	54/50 (108%)	31	23
Retained at follow‐up, n/N (%)	48/54 (88.9%)	31/31 (100.0%)	17/23 (73.9%)
Adherence frequency rate, mean% (range)	‐	81% (5%–145%)	‐
Adherence frequency rate, <40%/40%–70%/>70%	‐	4 / 4 / 23	‐
Adherence duration rate, mean% (range)	‐	119% (14.8%–348.1%)	‐
Adherence duration rate, <40%/40%–70%/>70%	‐	2 / 6 / 23	‐
Acceptability (% agreement)	‐	93.1%	‐

Abbreviation: HD = Huntington's disease.

**TABLE 2:** Demographic and clinical background information for eligible participants

	Total HD	HD‐DRUM	Control	Control (retained)
Participants characteristics				
Group size, n	n = 54	n = 31	n = 23	n = 17
Females, n (%)	29 (53.7%)	17 (54.8%)	12 (52.2%)	8 (47.1%)
Age‐years, mean (SD)	47.8 (12.9)	50.6 (11.1)	43.9 (14.1)	41.4 (14.9)
Education‐years, mean (SD)	14.2 (2.7)	14.8 (2.8)	13.3 (2.5)	13.4 (2.6)
CAG repeat length, mean (SD)	42.0 (2.6)	41.3 (2.2)	43.3 (2.6)	43.6 (3.0)
TFC, mean (SD)	11.9 (1.3)	12.2 (1.2)	11.5 (1.4)	11.5 (1.4)
SDMT, median (IQR)	43 (30–57)	47.5 (35.8–58)	34 (26–45)	34 (23.5–47.5)
TMS, median (IQR)	9 (3 .0–17.8)	6.5 (2.25–16.5)	11 (3.75–18.5)	12 (4. 5–17.8)
IS, median (IQR)	100 (90–100)	100 (95–100)	95 (85–100)	95 (85–100)
MOCA, mean (SD)	25.3 (3.6)	26.2 (3.1)	24.1 (3.8)	24.1 (4.2)
HD‐ISS	n	n	n	N
No CAG available	3	0	3	2
CAG 36–39	6	6	0	0
Undefined	3	2	1	0
Indeterminate 0 vs 1	5	2	3	2
HD‐ISS = 0	4	2	2	2
HD‐ISS = 1	7	5	2	2
HD‐ISS = 2	9	6	3	1
HD‐ISS = 3	17	8	9	8

Abbreviations: HD‐ISS = Huntington's Disease Integrated Staging System; IS = Independent Scale; MOCA = Montreal Cognitive Assessment^76^ (assessed at baseline only to determine general cognitive state); SDMT = Symbol Digit Modality Test; TFC, Total Functional Capacity; TMS, Total Motor Score.

### Retention

No participants withdrew from the HD‐DRUM group but six participants from the control group (Table 1). Reasons for withdrawal are summarized in Figure 1.

### Adherence

Most participants (23/31) met the predefined 70% criterion, engaging with HD‐DRUM on at least 28 occasions for a total duration of ≥315 min; maximum engagement reached 58 sessions (27 h). Six participants showed moderate adherence (40–69%), while two showed minimal adherence (< 40%), including one participant with no meaningful engagement (two sessions, 1 h total).

### Acceptability

Overall, participants rated the HD‐DRUM app as enjoyable (94%), engaging (81%), and easy to use (93.5%), with most valuing the training content, musical elements, and research team support (87%). Around half reported improvements in hand coordination (55%). Suggestions for optimizing acceptability included the need for more personalized feedback, and technical refinements to the app (Supplemental Table 2).

### Effect on Cognitive Performance

Data from 27 participants (19 HD‐DRUM) were included in the PCA due to missing data. Eleven components were identified, explaining 84% of data variance (Supplemental Fig. 1). One extreme outlier was excluded from each of four components. For Cog‐PC11, with high loadings on response speed in congruent flanker trials (Fig. 2), the 95% CIs of the group effect size excluded zero. This effect reflected a larger facilitation effect in control participants at baseline rather than a drumming‐related change (See Supplementary Fig. 8).

**Figure 2:**
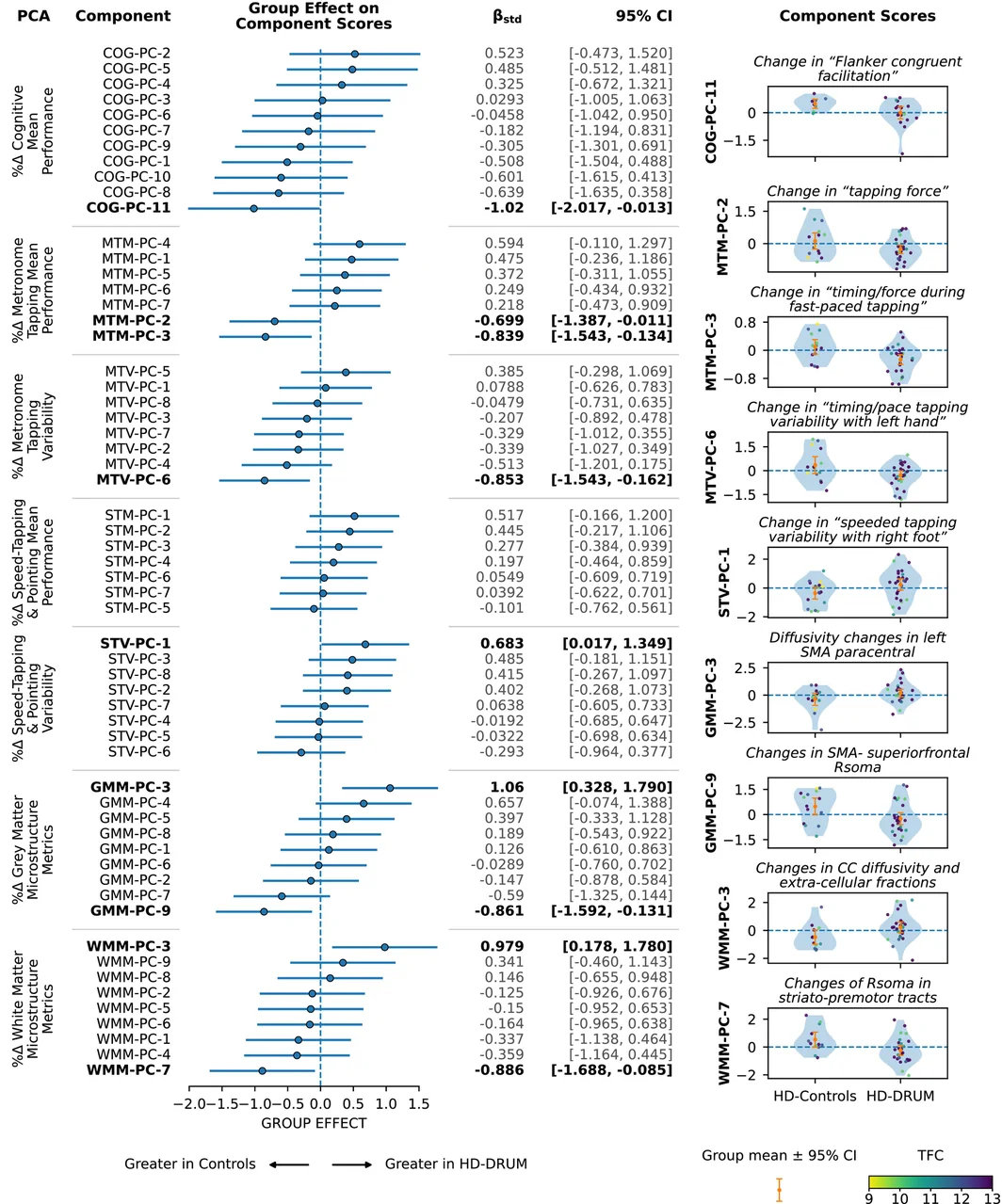
Effects of the HD‐DRUM intervention on cognitive performance, motor performance, and brain microstructure. Left: Forest plots of standardized between‐group effects (β_std_; 95% CI) on principal‐component (PC) scores from general linear models, derived from seven separate principal component analyses (PCAs) of percentage longitudinal change (%Δ) in outcome measures of Cognitive Performance (COG), of Metronome Tapping Mean Performance (MTM), of Metronome Tapping Variability (MTV), of Speeded‐Tapping and Pointing Mean Performance (STM), of Speeded‐Tapping and Pointing Variability (STV), of Grey‐Matter Microstructure (GMM), and of White‐Matter Microstructure (WMM). All models were adjusted for age and sex, and extreme outliers identified by the Tukey criterion (k = 3 × IQR) were excluded per component. The horizontal Group Effect axis is annotated such that positive βstd indicates higher PC scores in HD‐DRUM than in HD‐Controls. PCs whose 95% CI excludes zero are shown in bold and carried forward to the right column. Right: Violin plots of the score distributions of these selected PCs in HD‐Controls and HD‐DRUM, with individual participants overlaid, colour‐coded by Total Functional Capacity (TFC; range 9–13), and group mean ± 95% CI shown in orange. Descriptors above each violin plot summarise the variables based on the largest absolute rotated loadings on each PC.

### Effect on Motor Performance

Across the four Q‐Motor PCAs, seven or eight components were retained per analysis, explaining approximately 60% of the variance (Supplemental Fig. 2–7, Supplemental Table 3). Thirteen participants were identified as extreme outliers across up to two components each, resulting in 15 GLMs with one exclusion and two GLMs with two exclusions. For four components across three Q‐Motor PCAs, the 95% CIs of the group effect size excluded zero (Fig. 2). MTM‐PC2 and PC3 showed large group effects driven by longitudinal decreases in the HD‐DRUM group relative to controls. Salient loadings indicated improvements in tapping force (MTM‐PC2) and faster, more controlled timing and force during fast‐paced tapping (MTM‐PC3) following drumming. MTV‐PC6, characterized by left‐hand tapping measures, showed reduced variability after HD‐DRUM compared with increased variability in controls. Finally, STV‐PC1, loading on right‐foot speeded tapping, reflected reduced variability in controls but no change in the HD‐DRUM group; baseline differences indicated greater variability in controls that resolved at follow‐up. Baseline and follow‐up scores of the variables with the highest loading on each component are given in Supplementary Table 3.

### Effect on Mood

Mean (SD) PHQ‐8 scores were 4.29 (4.79) at baseline and 4.16 (4.38) at follow‐up in the HD‐DRUM group, and 7.00 (7.25) and 7.64 (7.80) in controls indicating no change in mood in either group.

### Effect on Brain Microstructure

GMM‐PCA included 41 participants (27 HD‐DRUM) and WMM‐PCA 38 participants (26 HD‐DRUM), due to failed tract reconstructions. Both PCAs retained nine components; one extreme outlier was excluded from each of six GLMs. Two GMM‐PCA components showed group effects with 95% CIs excluding zero (Fig. 2). GMM‐PC3, with loadings on left SMA–paracentral DTI and CHARMED metrics, was associated with decreased MD and RD and increased FA and FR in HD‐DRUM, with the opposite pattern in controls. GMM‐PC9, loading on bilateral SMA–superior frontal Rsoma, showed small decreases in HD‐DRUM versus increases in controls. Two WMM‐PCA components also showed group effects with 95% CIs excluding zero. WMM‐PC3, loading on central callosal diffusivity, ISOSF, and extracellular‐fraction metrics, reflected larger longitudinal decreases in controls and relative stability in HD‐DRUM. WMM‐PC7, loading on Rsoma in callosal genu and rostral body and bilateral striato‐prefrontal and striato‐premotor tracts, increased in controls but not in HD‐DRUM. Thus, HD‐DRUM‐related signals were observed in SMA regions, central/anterior CC, and striato‐prefrontal/premotor tracts. The structures and metrics carrying these four components, and the direction of change within each group, are shown in Supplementary Figure 9.

### Correlations between Motor and Brain Microstructure Changes

Correlation coefficients were calculated between the Q‐Motor (MTM‐PC2, MTM‐PC3, MTV‐PC6, STV‐ PC1) and brain microstructure components (GMM‐PC3, GMM‐PC9, WMM‐PC3, WMM‐PC7) that showed group ES with 95% CIs excluding zero (Fig. 3). In the HD‐DRUM group (*n* = 24), higher scores on MTV‐PC6 (left‐hand tapping variability increase, right‐hand decrease) were negatively associated with GMM‐PC9 (SMA–superior‐frontal Rsoma) (Pearson's *r* = −0.46, *P* = 0.02; Spearman's ρ = −0.45, *P* = 0.03). No significant correlations were observed in the control group.

**Figure 3:**
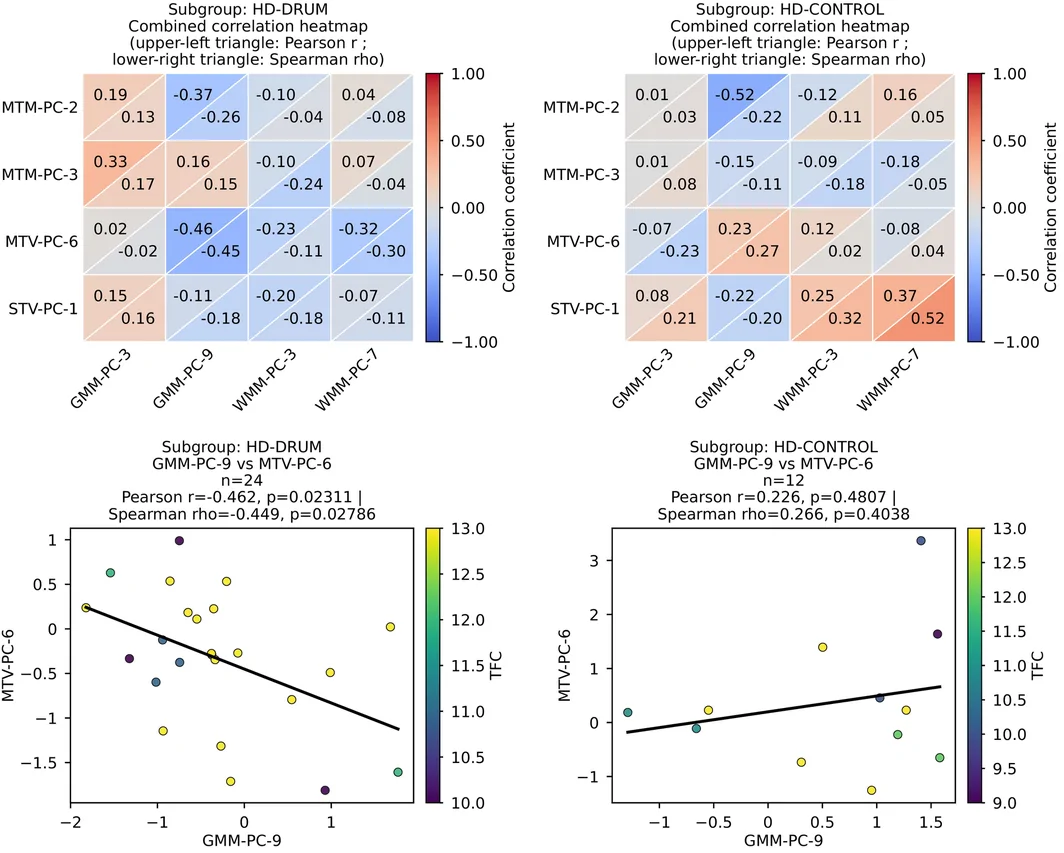
Post‐hoc correlations between motor and brain microstructure changes. Top: Combined correlation heatmaps between the four Q‐Motor (rows; MTM‐PC‐2 and MTM‐PC‐3 from Metronome Tapping Mean Performance, MTV‐PC‐6 from Metronome Tapping Variability, and STV‐PC‐1 from Speeded‐Tapping and Pointing Variability) and four brain‐microstructure (columns; GMM‐PC‐3 and GMM‐PC‐9 from Grey‐Matter Microstructure, WMM‐PC‐3 and WMM‐PC‐7 from White‐Matter Microstructure) principal‐component scores selected by the prior GLMs (95% CI excluding zero), shown separately for HD‐DRUM (left) and HD‐Controls (right). Each cell is split into Pearson r (upper‐left triangle) and Spearman ρ (lower‐right triangle), both colour‐coded on the same [−1, 1] scale. Computations used pairwise‐complete observations after per‐variable masking of extreme outliers (Tukey k = 3 × IQR). Bottom: Scatter plots of the only pair reaching *P* < 0.05, MTV‐PC‐6 versus GMM‐PC‐9, in HD‐DRUM (left; *n* = 24; Pearson *r* = −0.46, *P* = 0.023; Spearman ρ = −0.45, *P* = 0.028) and HD‐Controls (right; *n* = 12; Pearson *r* = 0.23, *P* = 0.48; Spearman ρ = 0.27, *P* = 0.40), with linear fits overlaid. Abbreviations: GMM, Grey‐Matter Microstructure; HD, Huntington's disease; IQR, interquartile range; MTM, Metronome Tapping Mean Performance; MTV, Metronome Tapping Variability; PC, principal component; STV, Speeded‐Tapping and Pointing Variability; WMM, White‐Matter Microstructure.

## Discussion

This pilot RCT evaluated the feasibility and explored preliminary effects of an 8‐week, home‐based, tablet‐delivered rhythmic movement training program (HD‐DRUM) compared with a usual‐activity control in individuals with HD. The primary objective was to determine whether a larger effectiveness trial would be achievable, assessed through recruitment, retention, adherence, and user acceptability, benchmarked against a predefined feasibility threshold of 70%.

All feasibility targets were met. Recruitment exceeded the planned sample size of 50 within the 24‐month recruitment window, and overall adherence and retention were high (83%), with no drop‐outs in the HD‐DRUM group. Participants rated the intervention as highly enjoyable, engaging, and easy to use, with most appreciating the musical content and support provided by the research team. These findings demonstrate that digitally delivered, home‐based rhythmic movement training is practical and acceptable for people with HD, and that recruitment, adherence, and retention in a future effectiveness RCT are feasible.

Although participants did not perceive improvements in cognition or mood over the 8‐week period, more than half reported improvements in hand coordination. Participant feedback highlighted challenges with training sessions involving irregular rhythms, bimanual coordination, or reversals, and technical issues such as freezing or reduced responsiveness. Suggested improvements focused on better tailoring of training difficulty and clearer, more personalized in‐app performance feedback to support progress monitoring. These insights are pivotal for optimizing the intervention prior to larger‐scale evaluation.

The study also explored HD‐DRUM‐related signals on motor performance, cognition, mood, and brain microstructure to inform outcome selection and potential mechanisms for a future RCT. Exploratory Q‐Motor analyses showed improvements in metronome‐paced tapping, particularly in components related to timing accuracy, force control, and performance consistency during fast‐paced tapping. Improvements were particularly apparent in the left, non‐dominant hand for most participants (29/31 were right‐handed), consistent with the non‐dominant hand's less developed baseline motor control,^77^ affording greater scope for training benefits. No improvements were observed for speeded and dual‐task tapping, cognition, or mood. These findings suggest that 8‐weeks of HD‐DRUM training specifically enhanced paced motor control abilities such as rhythmic sensorimotor synchronization, rather than leading to transfer benefits to general motor control, cognition, or mood.

Exploratory neuroimaging findings provided converging evidence for potential neural mechanisms supporting these behavioral effects. HD‐DRUM–related microstructural changes were observed in cortical and subcortical structures, including the SMA and paracentral cortices and striato‐prefrontal and striato‐premotor tracts. HD‐DRUM‐training was associated with relative maintenance or improvements in microstructural indices, whereas controls tended to show changes consistent with disease progression. Importantly, SMA microstructural changes correlated with changes in paced tapping variability, suggesting a link between training‐related neural plasticity and improved rhythmic motor control.

These observations are encouraging, as the implicated regions are central to beat perception, predictive timing, and motor coordination.^78^, ^79^ Previously we observed elevated apparent RSoma in the striatum of people with HD compared to healthy controls.^80^ Against this background, the relative maintenance of apparent RSoma in the HD‐DRUM group raises the possibility that rhythmic training may help slow microstructural decline in motor networks.

As a feasibility study, the trial was not designed or powered to assess clinical efficacy or long‐term disease modification. The absence of observed effects on cognition and mood contrasts with earlier uncontrolled pilot work,^22^, ^23^ likely reflecting the short intervention duration, low baseline mood symptoms, and the importance of controlling for practice effects. In progressive conditions such as HD, benefits may manifest as slowed decline rather than short‐term improvement, requiring longer follow‐up to detect meaningful effects.

Several considerations emerged for future trials, which were beyond the scope of this feasibility study. Only 35% of eligible individuals enrolled, partly due to competition with other clinical trials and time constraints. Potential confounds of medication, cognitive status, sensory impairment, and last clinical assessment before enrolment were not controlled for and some group differences may have influenced exploratory outcomes and should be addressed in future designs: participants were not equally allocated to the groups, and the control group was younger and had slightly worse cognitive and motor scores. Drop‐outs occurred in the control group, suggesting that an active control activity with more engagement to improve retention should be considered. A fully‐powered trial should also include clinical measures such as the UHDRS‐TMS as outcomes and assess participants over longer periods of time.

In conclusion, HD‐DRUM appears feasible, acceptable, and engaging as a home‐based rhythmic movement intervention for people with HD. Preliminary findings suggest specific improvements in rhythmic motor control and associated brain microstructure within beat‐perception networks. A fully powered RCT with an active control, prespecified primary outcomes, longer follow‐up, and an optimized intervention is warranted to determine clinical effectiveness, durability of effects, and impact on disease progression and quality of life.

## Author Roles

(1) Research Project: A. Conception, B. Organization, C. Execution; (2) Statistical Analysis: A. Design, B. Execution, C. Review and Critique; (3) Manuscript Preparation: A. Writing of the First Draft, B. Review and Critique.

P.L.L.: 2A, 2B, 2C, 3A, 3B

V.I.: 1B, 1C, 3B.

C.J.G.D: 1A, 1B, 3B.

P.P.: 1A, 2A, 2C, 3B.

M.B.M.: 1A, 3B.

G.B.W.: 1B, 3B.

R.S.: 1B, 2A, 2C, 3B.

A.E.R: 1A, 2B, 3B.

C.M.B.: 1A, 1B, 2A, 2B, 2C, 3A, 3B.

## Disclosures

**Ethical Compliance Statement:** This study received ethical approval from the NHS Health Research Authority Wales Research Ethics Committee 2 (REC reference: 22/WA/0147) and was sponsored by Cardiff University (SPON1895‐22). All participants provided written informed consent prior to taking part in the study. We confirm that we have read the Journal's position on issues involved in ethical publication and affirm that this work is consistent with those guidelines.

**Financial Disclosures and Conflicts of Interest:** The authors have no conflict of interest to declare. Author disclosures are available in the Supporting Information.

## Supporting information


**Supplemental Figure 1** Bubble grid of component loadings for the Cognitive Performance PCA. Rows show total number correct and response time variables included in the Cognitive Performance PCA and columns show retained principal components. Circle area is proportional to the absolute loading magnitude, and color indicates loading sign and magnitude (red = positive; blue = negative), highlighting variables that contribute most strongly to each component.
**Supplemental Figure 2** Bubble grid of component loadings for the Metronome Tapping Performance PCA. Rows show “means” variables included in the Metronome Tapping Performance PCA and columns show retained principal components. Circle area is proportional to the absolute loading magnitude, and color indicates loading sign and magnitude (red = positive; blue = negative), highlighting variables that contribute most strongly to each component.
**Supplemental Figure 3** Bubble grid of component loadings for the Metronome Tapping Variability PCA. Rows show “SD” variables included in the Metronome Tapping Variability PCA and columns show retained principal components. Circle area is proportional to the absolute loading magnitude, and color indicates loading sign and magnitude (red = positive; blue = negative), highlighting variables that contribute most strongly to each component.
**Supplemental Figure 4** Bubble grid of component loadings for the Speeded Tapping and Pointing Performance PCA. Rows show “mean” variables included in the Speeded Tapping and Pointing Variability PCA and columns show retained principal components. Circle area is proportional to the absolute loading magnitude, and color indicates loading sign and magnitude (red = positive; blue = negative), highlighting variables that contribute most strongly to each component.
**Supplemental Figure 5** Bubble grid of component loadings for the Speeded Tapping and Pointing Variability PCA. Rows show “SD” variables included in the Speeded Tapping and Pointing Variability PCA and columns show retained principal components. Circle area is proportional to the absolute loading magnitude, and color indicates loading sign and magnitude (red = positive; blue = negative), highlighting variables that contribute most strongly to each component.
**Supplemental Figure 6** Bubble grid of component loadings for the gray matter microstructural PCA. Rows show the gray matter microstructural variables included in the PCA and columns show retained principal components. Circle area is proportional to the absolute loading magnitude, and color indicates loading sign and magnitude (red = positive; blue = negative), highlighting variables that contribute most strongly to each component.
**Supplemental Figure 7** Bubble grid of component loadings for the white matter microstructural PCA. Rows show the white matter microstructural variables included in the PCA and columns show retained principal components. Circle area is proportional to the absolute loading magnitude, and color indicates loading sign and magnitude (red = positive; blue = negative), highlighting variables that contribute most strongly to each component.
**Supplemental Figure 8** Congruent‐flanker facilitation and its percentage change in the participants entering the cognitive PCA (HD‐Controls n = 8; HD‐DRUM n = 19). Response facilitation (mean reaction time on congruent flanker trials minus mean reaction time on single‐arrow trials, ms) at baseline and 8‐week follow‐up; group mean ± SEM with individual participants shown faintly. More negative values indicate greater facilitation.
**Supplemental Figure 9** Direction of regional microstructural change within each group, for the four microstructure components on which the groups differed. Panels show gray‐matter components GMM‐PC3 (A) and GMM‐PC9 (B) and white‐matter components WMM‐PC3 (C) and WMM‐PC7 (D). Rows are anatomical structures, columns are microstructure metrics, and a cell is drawn wherever that structure–metric variable loaded on the component at |loading| ≥ 0.50, the salience threshold used throughout. Blank cells are structure–metric variables that were measured but loaded below 0.50 on that component; structures and metrics with no salient loading on a component are not shown. Each cell is split diagonally: the upper‐left half is the HD‐DRUM group and the lower‐right half the HD‐Controls. Color gives the direction and approximate size of the mean percentage change from baseline to follow‐up within that group (red, increase; blue, decrease; three shades for <1%, 1–5% and ≥5%). (*) marks a within‐group mean differing from zero at *P* < 0.05, uncorrected; across the 122 tests shown these are descriptive, and one—radial diffusivity of the posterior callosal midbody in HD‐Controls—survives Benjamini–Hochberg correction. The sign at the top right of each cell is the direction of that variable's loading on the component. Gray‐matter panels use the 41 participants entering the gray‐matter PCA (27 HD‐DRUM, 14 HD‐Controls) and white‐matter panels the 38 entering the white‐matter PCA (26 HD‐DRUM, 12 HD‐Controls). Within‐group change and the between‐group difference are distinct quantities; the between‐group effects are reported in Figure 2 and are not shown here. Cortical labels are the Desikan–Killiany paracentral and superior frontal regions covering the SMA and the precentral gyrus; CC_2–CC_6 are parts of the seven‐part callosal parcellation; ST_PREF and ST_PREM are the striato‐prefrontal and striato‐premotor bundles. The brain maps above each grid show where its structures lie, with letters matching the grid rows (gray matter: FreeSurfer average brain, fsaverage; white matter: TractSeg bundles of one participant registered to MNI152 space); direction and size of change are shown only in the grids.
**Supplemental TABLE 1** Overview of the thematic components extracted with Principal Component Analysis and their key items of the acceptability questionnaire with component loadings and proportion of participant agreement.
**Supplemental TABLE 2** Representative quotes from thematic analysis of participant's qualitative feedback.
**Supplemental TABLE 3** Baseline and follow‐up scores of the highest‐loading variable of each retained principal component.

**Data S1** Supporting Information.

**Data S2** ICMJE Disclosure Statement Form.

## Data Availability

This project was endorsed by the Enroll‐HD Scientific Oversight Committee (SOC) (14/11/2022) and used Enroll‐HD captured clinical data. At the end of the project, the coded participant level data will be shared and made accessible through the Enroll‐HD specific data request process (enroll-hd.org).
