# Supplementary material for: HD‐DRUM Rhythmic Training in Huntington's Disease: Feasibility, Motor, and Brain Microstructural Pilot Signals
Source: Mov Disord Clin Pract. 2026 Oct 23:10.1002/mdc3.70871. Online ahead of print. doi: 10.1002/mdc3.70871 (PMC13691659; doi:10.1002/mdc3.70871)
Supplement: Supplementary file 1 [file MDC3-9999-0-s003.pdf]

This questionnaire aims to find out about your experiences with the drumming training app. Now that you have completed the study, we would like to ask you some questions about your experiences, how things may have changed for you as a result of being in this study and whether you have any feedback to us about the training.

For the following questions, please provide your level of agreement:

(1) strongly disagree to (5) strongly agree.

Strongly Disagree (1)---Disagree (2)---Neither Disagree/Agree (3)---Agree (4)---Strongly Agree (5)---Not applicable (6)

1. Overall, I enjoyed using the app and learning to drum along to different rhythms.
2. Overall, I felt motivated doing the training.
3. I found it easy to use the app.
4. I found it easy to tap on the tablet.
5. I would have preferred drumming on real Bongo drums.
6. I felt satisfied with the pace of the drumming training app.
7. I felt that I was training at the right level of difficulty for me, i.e., not too easy and not too hard.
8. Overall, I found the drumming training engaging.
9. Overall, I liked the different music rhythm and styles.
10. Overall, I felt that I received an adequate amount of feedback and encouragement from the app.
11. Overall, I feel that my ability to concentrate has improved since using the app.
12. Overall, I feel that my ability to multi-task has improved since using the app.
13. Overall, I feel my hand co-coordination has improved since using the app.
14. Overall, I feel my mood has improved since using the app.
15. Overall, I feel my motivation has improved since using the app.
16. I found the weekly check-ins with the research team useful to motivate me.
17. The researchers were responsive to my questions and concerns.
18. I received adequate instructions and assistance with the drumming training app from the researchers.
19. My family member(s) / carer(s) supported me with my drumming training programme.
20. Please provide information about how your family member/carer helped support your drumming training program (e.g. reminding me to do my drumming, taking part in the study as control participants, helping me to find the time to do the drumming).
    ……………………………………………………………………………………………………
    ……………………………………………………………………………………………………
    ……………………………………………………………………………………………………
    ……………………………………………………………………………………………………
    ……………………………………………………………………………………………………
    ………………………………………………………………………………
21. Overall, I was satisfied with the drumming training sessions.
22. I would have liked more feedback from the app about my drumming improvements.
23. I had to adapt my activities during the research programme to make time for the drumming training.
24. I found it easy to fit in the drumming training.
25. I was able to monitor my improvements throughout the programme.

26. I would like to continue with my drumming training after the research study finishes.
27. Please tell us how we could make the drumming training app better for you:
……
……
……
……
……
………………………………………………………………………

28. Please tell us what you liked/disliked about the drumming training app:
……
……
……
……
……
………………………………………………………………………

29. Please tell us about any struggles you might have encountered in using the drumming training app:
……
……
……
……
……
………………………………………………………………………

30. Please tell us anything else you would like to mention:
……
……
……
……
……
………………………………………………………………………
