# Supplementary material for: HD‐DRUM Rhythmic Training in Huntington's Disease: Feasibility, Motor, and Brain Microstructural Pilot Signals
Source: Mov Disord Clin Pract. 2026 Oct 23:10.1002/mdc3.70871. Online ahead of print. doi: 10.1002/mdc3.70871 (PMC13691659; doi:10.1002/mdc3.70871)
Supplement: Supplementary file 3 [file MDC3-9999-0-s002.docx]

Supplemental Figure 1

Supplemental Figure 2

Supplemental Figure 3

Supplemental Figure 4

Supplemental Figure 5

Supplemental Figure 6

Supplemental Figure 7

Supplemental Figure 8

Supplemental Figure 9

Supplemental Table 1

| **Components** | **Key Items** | **Component loading** | **% Participant agreement** |
| --- | --- | --- | --- |
| 1. User Engagement & Enjoyment | I enjoyed using the app. | 0.74 | 93.5 |
|  | I felt motivated doing the training. | 0.74 | 87.1 |
|  | I felt satisfied with the pace of the training. | 0.85 | 74.2 |
|  | I felt that I was training at the right level of difficulty for me. | 0.78 | 67.7 |
|  | I found the drumming training engaging. | 0.85 | 80.6 |
|  | I was satisfied with the drumming training sessions. | 0.82 | 83.9 |
| 2. Cognitive, Motor and Emotional Benefits | I feel that my ability to concentrate has improved. | 0.88 | 48.4 |
|  | I feel that my ability to multi-task has improved. | 0.86 | 29.0 |
|  | I feel that my hand coordination has improved. | 0.67 | 54.8 |
|  | I feel that my mood has improved. | 0.76 | 38.7 |
|  | I feel that my motivation has improved. | 0.76 | 38.7 |
| 3. Research Team Support | Researchers were responsive to my questions and concerns. | 0.94 | 87.1 |
|  | I received adequate instructions and assistance with the drumming training app. | 0.92 | 90.3 |
|  | I found it easy to use the app | 0.59 | 93.5 |
|  | I found the weekly check-ins useful to motivate me. | 0.51 | 64.5 |
| 4. Feedback and Monitoring | I would have liked more feedback from the app about my drumming improvements. | -0.78 | 76.7 |
|  | I was able to monitor my improvements throughout the programme. | 0.85 | 50.0 |
| 5. App Interaction | I found it easy to tap on the tablet. | 0.78 | 93.5 |
| 6. Activity Burden | I had to adapt my activities during the research programme to make time for the training. | -0.83 | 58.1 |
| 7. Music and Rhythm Appreciation | I liked the different music rhythm and styles. | -0.72 | 77.4 |
|  | I would like to continue with my drumming training after the research study finishes. | 0.55 | 56.7 |
| 8. Real Drumming Preference | I would have preferred drumming on real Bongo drums. | 0.81 | 22.6 |

Supplemental Table 2.

| Theme | Quotes | Notes |
| --- | --- | --- |
| 1. Enjoyment | - “Really enjoyed the drum app, I would have played from the app like that for fun outside of research reasons.” - “Intuitive, interactive and fun too.” - “Really enjoyed it and found it a great way to unwind before bed.” - “I liked learning how to drum in rhythm.” | **Enjoyment was related to** frequently expressed positive experiences of enjoyment and engagement with the drumming training. |
| 2. Feedback | - “It would have been nice to know my score when progressing to the next level.” - “Having feedback post session, even as a percentage of achievement/accuracy to motivate.” - “I would like to be able to see my results.”“Lack of feedback was an issue for me.” | **Feedback-related statements expressed participants’ wishes for more performance feedback and clarity of progress within the app, such as** |
| 3. Difficulty | - “Sessions 8, 16, 17 were hard.” - “I got stuck on session 6 after about 1 week and it took several weeks to get through it.” - “Left to right coordination was fine, but left-left-right-left-left-right-left-right I struggled with.”   • “I found it hard from session 5.” | **Participants often mentioned challenges with specific training sessions or coordination:** |
| 4. Technical issues | - “The app kept freezing on session, so had to reboot a number of times.” - “Sometimes when doing fast drumming the tablet itself could not keep up with the screen inputs.” - “The screen didn’t pick up all my strikes.” - “Better response from the tablet. It felt too delayed sometimes.” | **App technical issues affected user experience in different ways** |
| 5. Support | - “My wife helped me during session 5 by showing me how it’s done.” - “My daughter texted the researchers when I had a query and the problem was quick-ly resolved.” - “My son and daughter supported me and encouraged me throughout.” - “My family asked about how it was going. They thought it was an important thing to take part in.” | **Support from family or researchers was appreciated and often mentioned** |
| 6. Suggestions | - “It would be nice to have practice Bongos to mess about with outside of the ses-sions.” - “Once you have done a level once, but not completed it, it would be nice not to have to play through all of the talkie bits over and over.” - “A paper printout showing how the rhythm and beats work would help.” - “You should be able to choose from more music genres.” | **Participants offered a number of ideas for improvement:** |

Supplemental Table 3

| **Component** | **Variable with highest loading** | **Measure** | **Loading** | **HD-DRUM** | | **HD Control** | |
| --- | --- | --- | --- | --- | --- | --- | --- |
|  |  |  |  | **Baseline** | **Follow-up** | **Baseline** | **Follow-up** |
| **Cognition** | | | | | | | |
| COG-PC1 | flanker2 | Eriksen flanker (PEBL): mean reaction time, all trials (ms) | +0.972 | 599.5 (218.4) *n = 31* | 567.8 (201.1) *n = 31* | 641.7 (248.5) *n = 21* | 593.8 (228.0) *n = 17* |
| COG-PC2 | vt7 | Verbal trails: letter–digit switch, time to complete (s) | +0.933 | 40.14 (15.74) *n = 28* | 40.37 (22.90) *n = 30* | 65.15 (36.17) *n = 20* | 42.57 (8.71) *n = 14* |
| COG-PC3 | stroop_c1 | Stroop, colour form: total correct (n) | +0.899 | 110.2 (5.3) *n = 30* | 110.9 (3.9) *n = 31* | 109.1 (8.1) *n = 22* | 104.4 (18.2) *n = 16* |
| COG-PC4 | nback1_dual_p_aprime | Dual-task position 1-back: A′ sensitivity | +0.831 | 0.867 (0.203) *n = 25* | 0.907 (0.127) *n = 27* | 0.823 (0.242) *n = 18* | 0.845 (0.196) *n = 13* |
| COG-PC5 | flanker9 | Eriksen flanker (PEBL): conflict cost, incongruent mean RT − congruent mean RT (ms) | +0.838 | 104.6 (55.9) *n = 31* | 69.77 (32.58) *n = 31* | 98.49 (49.86) *n = 21* | 85.97 (95.51) *n = 17* |
| COG-PC6 | vt3 | Verbal trails: letters baseline, time to complete (s) | +0.814 | 23.20 (9.61) *n = 30* | 21.20 (8.26) *n = 30* | 26.18 (10.99) *n = 22* | 23.12 (7.36) *n = 16* |
| COG-PC7 | nback1_a_aprime | Auditory 1-back: A′ sensitivity | +0.700 | 0.992 (0.018) *n = 31* | 0.991 (0.018) *n = 30* | 0.970 (0.057) *n = 23* | 0.971 (0.062) *n = 17* |
| COG-PC8 | nback1_p_aprime | Position 1-back: A′ sensitivity | -0.830 | 0.889 (0.185) *n = 31* | 0.891 (0.220) *n = 30* | 0.852 (0.219) *n = 23* | 0.899 (0.178) *n = 17* |
| COG-PC9 | stroop_cw4 | Stroop interference, colour–word form: time to complete (s) | +0.842 | 113.4 (11.1) *n = 30* | 112.1 (13.2) *n = 31* | 114.9 (11.7) *n = 22* | 116.3 (9.5) *n = 17* |
| COG-PC10 | nback2_a_aprime | Auditory 2-back: A′ sensitivity | +0.945 | 0.898 (0.094) *n = 31* | 0.898 (0.088) *n = 30* | 0.818 (0.184) *n = 23* | 0.858 (0.209) *n = 17* |
| COG-PC11 | flanker14 | Eriksen flanker (PEBL): congruent mean RT − single-arrow mean RT (ms) | +0.871 | -15.10 (29.35) *n = 31* | -11.68 (30.33) *n = 31* | -24.94 (38.31) *n = 21* | -26.72 (56.14) *n = 17* |
| **Metronome Tapping — Mean Performance** | | | | | | | |
| MTM-PC1 | TMIOMNHR_PS | Metronome tapping: inter onset interval, mean, right hand, 1.1 s paced (s) | +0.939 | 1.04 (0.05) *n = 31* | 1.06 (0.03) *n = 31* | 1.02 (0.16) *n = 23* | 1.06 (0.04) *n = 17* |
| MTM-PC2 | TMACMNFR_US | Metronome tapping: area under the curve, mean, right foot, 1.1 s unpaced (Ns) | +0.860 | 2.99 (2.25) *n = 30* | 2.55 (2.40) *n = 29* | 2.25 (1.66) *n = 23* | 2.50 (2.52) *n = 16* |
| MTM-PC3 | TMIOMNFR_PF | Metronome tapping: inter onset interval, mean, right foot, 0.55 s paced (s) | +0.884 | 0.541 (0.048) *n = 30* | 0.535 (0.036) *n = 29* | 0.530 (0.052) *n = 23* | 0.554 (0.084) *n = 16* |
| MTM-PC4 | TMACMNHL_UF | Metronome tapping: area under the curve, mean, left hand, 0.55 s unpaced (Ns) | +0.815 | 0.220 (0.177) *n = 31* | 0.232 (0.231) *n = 31* | 0.344 (0.337) *n = 23* | 0.251 (0.253) *n = 17* |
| MTM-PC5 | TMACMNHR_UF | Metronome tapping: area under the curve, mean, right hand, 0.55 s unpaced (Ns) | +0.696 | 0.164 (0.162) *n = 31* | 0.158 (0.167) *n = 31* | 0.193 (0.174) *n = 23* | 0.159 (0.144) *n = 17* |
| MTM-PC6 | TMAPMNFR_US | Metronome tapping: absolute deviation from pace, mean, right foot, 1.1 s unpaced (s) | +0.686 | 0.123 (0.076) *n = 30* | 0.116 (0.063) *n = 29* | 0.207 (0.156) *n = 23* | 0.167 (0.126) *n = 16* |
| MTM-PC7 | TMACMNFR_UF | Metronome tapping: area under the curve, mean, right foot, 0.55 s unpaced (Ns) | +0.653 | 1.74 (1.25) *n = 30* | 1.51 (1.16) *n = 29* | 1.56 (1.65) *n = 23* | 1.43 (1.34) *n = 16* |
| **Metronome Tapping — Variability** | | | | | | | |
| MTV-PC1 | TMAPSDFR_PF | Metronome tapping: absolute deviation from pace, SD, right foot, 0.55 s paced (s) | +0.960 | 0.0689 (0.0439) *n = 30* | 0.0473 (0.0299) *n = 29* | 0.0700 (0.0378) *n = 23* | 0.0980 (0.1510) *n = 16* |
| MTV-PC2 | TMAPSDHR_PF | Metronome tapping: absolute deviation from pace, SD, right hand, 0.55 s paced (s) | +0.915 | 0.0502 (0.0271) *n = 31* | 0.0514 (0.0407) *n = 31* | 0.0527 (0.0320) *n = 23* | 0.0603 (0.0278) *n = 17* |
| MTV-PC3 | TMAPSDFR_UF | Metronome tapping: absolute deviation from pace, SD, right foot, 0.55 s unpaced (s) | +0.836 | 0.0546 (0.0556) *n = 30* | 0.0455 (0.0361) *n = 29* | 0.105 (0.143) *n = 23* | 0.0992 (0.1450) *n = 16* |
| MTV-PC4 | TMACSDHL_PS | Metronome tapping: area under the curve, SD, left hand, 1.1 s paced (Ns) | +0.834 | 0.168 (0.201) *n = 31* | 0.141 (0.144) *n = 31* | 0.227 (0.366) *n = 23* | 0.170 (0.272) *n = 17* |
| MTV-PC5 | TMIOSDHL_UF | Metronome tapping: inter onset interval, SD, left hand, 0.55 s unpaced (s) | +0.735 | 0.0534 (0.0373) *n = 31* | 0.0524 (0.0504) *n = 31* | 0.105 (0.153) *n = 23* | 0.0823 (0.0840) *n = 17* |
| MTV-PC6 | TMIOSDHL_PF | Metronome tapping: inter onset interval, SD, left hand, 0.55 s paced (s) | +0.784 | 0.0759 (0.0515) *n = 31* | 0.0674 (0.0324) *n = 31* | 0.0887 (0.0745) *n = 23* | 0.0868 (0.0782) *n = 17* |
| MTV-PC7 | TMAPSDFR_US | Metronome tapping: absolute deviation from pace, SD, right foot, 1.1 s unpaced (s) | -0.806 | 0.0829 (0.0647) *n = 30* | 0.0829 (0.0666) *n = 29* | 0.132 (0.097) *n = 23* | 0.109 (0.074) *n = 16* |
| MTV-PC8 | TMAPSDHR_UF | Metronome tapping: absolute deviation from pace, SD, right hand, 0.55 s unpaced (s) | -0.793 | 0.0340 (0.0276) *n = 31* | 0.0329 (0.0299) *n = 31* | 0.0475 (0.0324) *n = 23* | 0.0537 (0.0426) *n = 17* |
| **Speeded Tapping & Pointing — Mean Performance** | | | | | | | |
| STM-PC1 | TSITMNFR | Speeded tapping: inter tap interval, mean, right foot (s) | +0.832 | 0.207 (0.109) *n = 31* | 0.209 (0.130) *n = 30* | 0.236 (0.123) *n = 23* | 0.210 (0.089) *n = 17* |
| STM-PC2 | TSTDMNFR | Speeded tapping: tap duration, mean, right foot (s) | +0.841 | 0.143 (0.094) *n = 31* | 0.135 (0.073) *n = 30* | 0.152 (0.095) *n = 23* | 0.132 (0.077) *n = 17* |
| STM-PC3 | TSACMNFL | Speeded tapping: area under the curve, mean, left foot (Ns) | +0.896 | 1.35 (1.43) *n = 29* | 1.15 (1.03) *n = 28* | 1.58 (1.92) *n = 23* | 1.02 (0.82) *n = 17* |
| STM-PC4 | TSTDMNHL | Speeded tapping: tap duration, mean, left hand (s) | +0.915 | 0.111 (0.050) *n = 31* | 0.103 (0.041) *n = 31* | 0.138 (0.115) *n = 23* | 0.113 (0.057) *n = 17* |
| STM-PC5 | TSTDMNHR | Speeded tapping: tap duration, mean, right hand (s) | +0.820 | 0.0927 (0.0363) *n = 31* | 0.0895 (0.0335) *n = 31* | 0.101 (0.047) *n = 23* | 0.0960 (0.0409) *n = 17* |
| STM-PC6 | PTTDMNHD | Pointing and tapping: tap duration, mean, both hands (s) | +0.909 | 0.166 (0.099) *n = 31* | 0.159 (0.072) *n = 31* | 0.221 (0.188) *n = 23* | 0.202 (0.154) *n = 17* |
| STM-PC7 | TSIOMNHL | Speeded tapping: inter onset interval, mean, left hand (s) | +0.823 | 0.254 (0.106) *n = 31* | 0.248 (0.093) *n = 31* | 0.303 (0.197) *n = 23* | 0.271 (0.106) *n = 17* |
| **Speeded Tapping & Pointing — Variability** | | | | | | | |
| STV-PC1 | TSIOSDFR | Speeded tapping: inter onset interval, SD, right foot (s) | +0.897 | 0.100 (0.074) *n = 31* | 0.0889 (0.0617) *n = 30* | 0.156 (0.148) *n = 23* | 0.107 (0.075) *n = 17* |
| STV-PC2 | TSIOSDHR | Speeded tapping: inter onset interval, SD, right hand (s) | +0.925 | 0.0490 (0.0464) *n = 31* | 0.0447 (0.0404) *n = 31* | 0.0671 (0.0606) *n = 23* | 0.0627 (0.0664) *n = 17* |
| STV-PC3 | TSACSDHR | Speeded tapping: area under the curve, SD, right hand (Ns) | +0.799 | 0.0500 (0.0769) *n = 31* | 0.0387 (0.0453) *n = 31* | 0.0807 (0.1282) *n = 23* | 0.0583 (0.0805) *n = 17* |
| STV-PC4 | TSTDSDFL | Speeded tapping: tap duration, SD, left foot (s) | +0.876 | 0.0628 (0.0561) *n = 29* | 0.0532 (0.0428) *n = 28* | 0.0831 (0.0994) *n = 23* | 0.0602 (0.0461) *n = 17* |
| STV-PC5 | PTTDSDHD | Pointing and tapping: tap duration, SD, both hands (s) | +0.892 | 0.0864 (0.0987) *n = 31* | 0.0693 (0.0703) *n = 31* | 0.165 (0.256) *n = 23* | 0.113 (0.136) *n = 17* |
| STV-PC6 | TSIOSDHL | Speeded tapping: inter onset interval, SD, left hand (s) | +0.899 | 0.0597 (0.0458) *n = 31* | 0.0535 (0.0367) *n = 31* | 0.0972 (0.1246) *n = 23* | 0.0833 (0.0879) *n = 17* |
| STV-PC7 | PTIPSDHD | Pointing and tapping: inter peak interval, SD, both hands (s) | +0.806 | 0.158 (0.128) *n = 31* | 0.142 (0.134) *n = 31* | 0.270 (0.228) *n = 23* | 0.176 (0.128) *n = 17* |
| STV-PC8 | TSAOSDHL | Speeded tapping: absolute onset, SD, left hand (s) | -0.741 | 2.71 (0.07) *n = 31* | 2.74 (0.05) *n = 31* | 2.70 (0.09) *n = 23* | 2.72 (0.10) *n = 17* |

Values are means (standard deviations) of the raw score at each timepoint, with the number of participants contributing to each cell. One row is shown per component retained in the five behavioural principal component analyses (PCAs): cognition (COG) and the four Q-Motor analyses — metronome tapping mean performance (MTM) and variability (MTV), speeded tapping and pointing mean performance (STM) and variability (STV). The variable shown is the one with the largest absolute rotated loading on that component; the Measure column expands its code, with the unit of the tabulated values in parentheses.

Sample: all analysed gene-expansion carriers (31 HD-DRUM, 23 control). Cell sizes differ because of missing individual assessments and, at follow-up, because six control participants were lost to follow-up. Scores are unadjusted; no outlier exclusion was applied, unlike the component-score general linear models in Figure 2.

Loadings are taken from the Varimax-rotated component matrices and refer to the percentage change of that variable from baseline to follow-up (named …_pDELTA there); the sign gives the direction of the variable’s contribution to its component.

Q-Motor codes: TM = metronome tapping, TS = speeded tapping, PT = pointing; MN = mean and SD = standard deviation across trials; IO = inter-onset interval (higher = slower tapping); FL/FR = left/right foot, HL/HR = left/right hand; PF/PS = fast-/slow-paced, UF/US = unpaced fast/slow. The codes AC, AO, AP, IP, IT, TD and HD are internal Q-Motor labels and are not expanded here.
